# Countering adipose tissue dysfunction could underlie the superiority of telmisartan in the treatment of obesity-related hypertension

**DOI:** 10.1186/s12933-021-01259-w

**Published:** 2021-03-24

**Authors:** Yahya M. Naguib, Rehab M. Samaka, Mohamed S. Rizk, Omnia Ameen, Shaimaa M. Motawea

**Affiliations:** 1 Physiology Department, College of Medicine and Medical Sciences, Arabian Gulf University, Manama, Bahrain; 2 Clinical Physiology Department, Faculty of Medicine, Menoufia University, Menoufia, Egypt; 3 Pathology Department, Faculty of Medicine, Menoufia University, Menoufia, Egypt; 4 Medical Biochemistry and Molecular Biology Department, Faculty of Medicine, Menoufia University, Menoufia, Egypt

**Keywords:** Obesity, Hypertension, Angiotensin receptor blockers, Telmisartan, Adipose tissue

## Abstract

**Background:**

The prevalence of hypertension and obesity has increased significantly in recent decades. Hypertension and obesity often coexist, and both are associated with increased cardiovascular mortality. Obese hypertensive patients usually require special anti-hypertensive treatment strategy due to the increased risk of treatment resistance. Molecules that can target both obesity and hypertension underlying pathologies should get more attention. Herein, we evaluated the therapeutic effects of telmisartan, with special interest in visceral adipose tissue dysfunction, in obesity-related hypertension rat model.

**Methods:**

Thirty male Wistar rats weighing 150–200 g were equally divided into: 1—Control group (fed normal laboratory diet for 24 weeks), 2—Diet-induced obesity group (DIO, fed high fat diet for 24 weeks), and 3—Diet-induced obesity treated with telmisartan group (DIO + Tel, fed high fat diet and received telmisartan for 24 weeks). At the end of the study, anthropometrical parameters were evaluated. Systolic blood pressure and heart rate were measured. Blood samples were collected for the measurement of serum lipids, adipokines, cardiac, renal, inflammatory, and oxidative stress biomarkers. Kidneys were removed and used for histopathological studies, and visceral adipose tissue was utilized for histopathological, immunohistochemical and RT-PCR studies.

**Results:**

High fat diet resulted in obesity-related changes in anthropometrical parameters, elevation of blood pressure, increase in heart rate, higher serum levels of cardiac, inflammatory and kidney function biomarkers, with altered serum lipids, adipokines and oxidative stress markers. Morphological changes (H&E and PAS-stained sections) were noticed in kidneys and visceral adipose tissue. Immunohistochemistry and RT-PCR studies confirmed adipose tissue dysfunction and over-expression of inflammatory and oxidative stress proteins. Telmisartan countered obesity-induced alterations in cardiovascular, renal, and adipose tissue functions.

**Conclusion:**

Adipose tissue dysfunction could be the core pathophysiology of obesity-related hypertension. Besides its anti-hypertensive effect, telmisartan had profound actions on visceral adipose tissue structure and function. Attention should be given to polymodal molecules targeting adipose tissue-related disorders.

## Background

Obesity associated comorbidities have become an increasing major health risk as well as a huge burden on health systems [1]. Hypertension and associated cardiometabolic disorders are unfavoured major outcomes of obesity or even overweight. The relation between body mass index (BMI) and systolic blood pressure (SBP) or diastolic blood pressure (DBP) is almost linear [2, 3]. Adipose tissue distribution has a remarkable impact; obesity associated with increased visceral adiposity is considered as a major cause of hypertension and accounts for almost three-fourth of the risk of developing essential hypertension. In fact, visceral fat seems to be better predictor of elevated blood pressure (BP) than subcutaneous fat [4]. In fact, adipose tissue is now considered as an endocrine organ and an active tissue for energy homeostasis, and not only an inert energy storage tissue. Adipose tissue synthesises and secretes hormones (e.g., leptin and adiponectin) and inflammatory mediators (e.g., TNF-α and IL-6), and controls insulin sensitivity [5, 6]. Obesity induces adipose tissue dysfunction. Adipose tissue dysfunction represents tissue remodelling characterized by adipocyte hypertrophy and hyperplasia, increased secretion of pro-inflammatory adipokine, inflammatory cell infiltration, mitochondrial dysfunction, and tissue inflammation [5, 7, 8].

In most of the cases, the impairment of pressure natriuresis, secondary to the increase in the renal tubular sodium reabsorption, is a vital event in initiating obesity hypertension [9]. Several obesity-related factors could disrupt the renal-pressure natriuresis and cause hypertension. Physical compression of the kidneys by increased visceral, retroperitoneal, and renal sinus fat presents as a major factor [10]. Another central factor is the activation of sympathetic nervous system (SNS), especially the renal sympathetic nerve activity (RSNA), Activation of the SNS can be partially explained by the leptin hormone and the activation of the brain melanocortin system [11]. Another important factor contributing to the impairment of pressure natriuresis in obese hypertensive patients is the activation of the renin–angiotensin–aldosterone system (RAAS). Multiple mechanisms contribute to the activation of the RAAS in obesity including renal compression by visceral fat and increased SNS activation [12].

Obesity-dependent hypertension is mainly related to visceral adiposity, which is mostly associated with dysfunction of adipose tissue, dyslipidemia, and insulin resistance. Therefore, an efficient therapeutic approach for obesity-related hypertension should cover both the elevated blood pressure as well as the obesity related metabolic disorders [13]. Several studies suggested that angiotensin receptors blockers (ARBs) could improve adipocytes differentiation, attenuate the inflammatory process in adipose tissue, and improve adipocytokine formation [14]. Although olmesartan inhibited adipocyte hypertrophy and suppressed IL-6 gene expression [15], it appeared ineffective in lowering serum leptin levels in clinical studies when compared to telmisartan [16]. Candesartan was also reported to guard against hypertension and adipocyte dysfunction in spontaneously hypertensive rats [17]. Valsartan reduced abdominal subcutaneous adipocyte size as well as adipose tissue macrophage infiltration markers [18].

Previous human and animal studies have demonstrated that telmisartan, an ARB and a partial agonist of peroxisome proliferator-activated receptor gamma (PPAR-γ), can improve obesity related cardiometabolic sequels in obesity hypertension [19, 20]. In patients with diabetes and high cardiovascular risk who participated in the ONTARGET/TRANSCEND (telmisartan alone and in combination with ramipril) trials, a U-shaped relationship between SBP and adjusted cardiovascular diseases rates in patients with Type 2 diabetes mellitus (T2DM) and established cardiovascular disease or multiple risk factors has been demonstrated. SBP less than 120 mmHg was associated with higher risk for cardiovascular events and death [21, 22]. Previous reports demonstrated that telmisartan induced browning of the fully differentiated white adipocytes, at least in part, via PPAR-γ mediated M2 polarization [23]. Unfortunately, a specific role of cardiac adipose tissue in the development of CVD in diabetes could not be established [24]. The role of telmisartan in improving insulin resistance and energy homeostasis is well documented. Telmisartan improved insulin resistance, modulating adipose tissue macrophage polarization in high-fat-fed mice [25]. Telmisartan was also reported to prevent obesity by increasing the expression of uncoupling protein 1 in diet-induced obese mice [26]. The favorable effects of telmisartan on obesity was explained by its action as a partial agonist of PPAR-γ beyond its blood pressure-lowering effect [27]. However, candesartan, with much lower PPAR-γ, attenuated comparably weight-gain in an animal model of high fat diet-induced obesity [25].

Although the risk of obesity, especially when accompanied with increased visceral adiposity, as a major cause of elevated BP is fully established, the pathophysiological mechanisms are complicated and have not been fully clarified yet. In Addition, the underlying mechanisms explaining the precise role of ARBs in the treatment of obesity hypertension need to be explained. Therefore, the present study investigated the therapeutic effects of telmisartan in obese hypertensive rats and studied the possible underlying mechanisms for its cardiometabolic and renal effects.

## Methods

All experiments and animal care and use were approved by and in accordance with the guidelines of the Ethics Committee at the Faculty of Medicine Menoufia University. Experiments were conducted in adherence to the Guiding Principles in the Use and Care of Animals published by the National Institutes of Health (NIH Publication No 85–23, Revised 1996). Thirty male Wistar rats weighing 150–200 g were purchased from a local providing facility and recruited for the present study. Rats were maintained for 1 week to acclimatize and were given free access to normal diet and water in an air-conditioned room with a 12-h light–dark cycles. All efforts were made to minimize stress and pain and ensure animal welfare.

After being acclimatized, rats were divided randomly and equally (10 rats per group) into 3 experimental groups:

1. Control group: rats were fed standard laboratory diet (containing 23.5% protein, 63% carbohydrate and 13.5% fat), and received dimethyl sulfoxide (DMSO, Sigma-Aldrich Chemie GmbH, Germany) via gastric lavage for 24 consecutive weeks.
2. Diet induced obesity (DIO) group: rats were fed high fat diet (containing 7% protein, 37% carbohydrate and 56% fat) [28], and received DMSO via gastric lavage for 24 consecutive weeks. The source of fat was mainly lard.
3. Diet induced obesity + telmisartan (DIO + Tel) treated group: rats were fed high fat diet and received telmisartan (Sigma-Aldrich Chemie GmbH, Germany) dissolved in DMSO in a dose of 8 mg/kg/day via gastric lavage for 24 consecutive weeks [29].

At the end of the study, all rats were fasted overnight. The next day, blood pressure and heart rate were measured. Following that, rats were anaesthetized. Anthropometrical parameters were taken, blood samples were collected, and kidneys and visceral adipose tissue were removed. All rats were then sacrificed by cervical dislocation.

### Anthropometric measurements

Anthropometric parameters were determined in all rats. The abdominal circumference (AC) was measured immediately anterior to the forefoot. The rat body length was measured nose to anus. The rat body weight and body length were used to calculate the body mass index (BMI) (BMI = body weight (g)/length^2^ (cm^2^)) [30].

### Measurement of systolic blood pressure (SBP) and heart rate (HR)

Rat systolic blood pressure was measured as described previously [31]. Briefly, a rat-tail pressure detecting equipment (Harvard apparatus Ltd, Aden Berge, England) was connected to a pneumatic transducer (Harvard U.K.) and the changes in rat systolic blood pressure were recorded on a physiograph (MK III-S, Narco BioSystem, USA). A pulsed doppler flowmeter (Hadeco, Hayashi Denki Co. Ltd., Japan) was used to evaluate the rat heart rate (HR) as described previously [31].

### Collection of blood samples

Rats were anaesthetised via intraperitoneal injection of sodium thiopental (STP, 60 mg/kg). Subsequently, blood samples were withdrawn via cardiac puncture. Blood was left undisturbed to clot at room temperature for 20–30 min. Blood was centrifuged (1000–2000 rpm for 10 min) to separate the serum. The collected serum samples were used immediately or stored at − 20 °C for further biochemical analysis.

### Biochemical analysis

Serum cholesterol, triglycerides, low density lipoproteins (LDL), very low density lipoproteins (VLDL), and high density lipoproteins (HDL) were measured by colorimetric kits (Sigma-Aldrich Inc, Saint Louis, Missouri, USA), using COBAS C 311 Automated Chemistry Analyzer (Roche, Basel, Switzerland). Serum ELISA kits for kidney injury molecule 1 (KIM-1), creatinine, lactate dehydrogenase (LDH), cardiac troponin I (cTnI), high sensitivity C reactive protein (hsCRP), tumour necrosis factor alpha (TNF-α), interleukin 6 (IL-6), superoxide dismutase (SOD), malondialdehyde (MDA), leptin, adiponectin, and ghrelin were purchased from MyBioSource (MyBioSource Inc, San Diego, CA, USA), while nitric oxide (NO) ELISA kit was purchased from Creative Diagnostics (CD Creative Diagnostics, NY, USA). An ELISA absorbance microplate reader (Infinite F50, Tecan Group Ltd., Switzerland) was used.

### Visceral adipose tissue and renal histopathology

Visceral adipose tissues and kidney were dissected and submitted to Pathology Department, Faculty of Medicine, Menoufia University. They were fixed in 10% neutral buffered formalin, dehydrated in ascending grades of ethanol followed by immersion in xylene then impregnated in paraffin. Five µm-thick sections from each block (Visceral fat and renal tissue) were taken and stained by haematoxylin and eosin (H&E) for routine histopathological examination. For renal tissue, Periodic Acid Schiff (PAS) staining was performed to assess the status of glomerular basement membrane with the PAS special stain [32]. Histopathological evaluation of the rat visceral adipose tissue was performed as described previously [33]. Adipocytes type (white or brown), uniformity, integrity and inflammatory cellular infiltrates were assessed. Histopathological evaluation of renal tissue including glomerular parameters (status, cellularity, lobulation, mesangial cell proliferation, mesangial matrix deposition, patency of capillaries, crescent formation, patency of bowman’s space), tubular and interstitial parameters (necrosis, cast formation, fibrosis, and inflammatory cellular infiltrates). An Olympus microscope BX53 with a digital camera was used for assessment and acquiring digital images.

### Visceral adipose tissue immunohistochemistry

Rat fat immunohistochemistry was performed as described previously [34]. Five µm-thick serial sections were cut from each paraffin block and mounted on positively charged glass slides to be ready for immunohistochemical staining using streptavidin–biotin amplified system. The primary antibodies were a rabbit polyclonal anti leptin (ready to use, cat. # sc-842, Santa Cruz, CA), a rabbit polyclonal anti- inducible nitric oxide synthase (iNOS) (ready to use, cat. # RB-9242 Thermo Scientific, Lab Vision Corporation, USA), and a mouse monoclonal anti-tumour necrosis alpha (TNF-α) (ready to use, cat. # sc-52746, Santa Cruz, CA, USA). Ultra V block was applied to block nonspecific background staining. Antigen retrieval was performed using Tris–EDTA (pH 9). An UltraVision Detection System Anti-polyvalent HRP/DAB (ready to use, cat. #TP-015-HD; Lab Vision Corporation, Fremont, California) was used. Finally, the reaction was visualized by appropriate substrate/chromogen (Diaminobenzidine, DAB) reagent with Mayer’s hematoxylin as a counterstain. The staining procedure included positive tissue control for leptin (breast carcinoma), iNOS (lung) and TNF-α (breast carcinoma) and negative tissue controls by omitting the primary antibodies.

### Quantitative RT-PCR (qRT-PCR)

The rat visceral adipose tissue was excised, weighed, and either stored at − 80 or used freshly for real time quantitative reverse transcription-polymerase chain reaction (RT-PCR) experiments. Primer Express Software version 3.0.1 (Applied Biosystems, USA) was utilized to design gene specific primers. The forward primer for leptin was (CCTGTGGCTTTGGTCCTATCTG), and the reverse primer was (AGGCAAGCTGGTGAGGATCTG). The forward primer for the inducible nitric oxide synthase (iNOS) was (CACCACCCTCCTTGTTCAAC), and the reverse primer was (CAATCCACAACTCGCTCCAA). The forward primer for TNF-α was (AAATGGGCTCCCTCTCATCAGTTC), and the reverse primer was (TCCGCTTGGTGGTTTGCTACGAC). The forward primer for β-actin was (TGTTTGAGACCTTCAACACC), and the reverse primer was (TAGGAGCCAGGGCAGTAATC). β-actin was used as the housekeeping control gene. All primers were ordered from Sigma-Aldrich (Chemie GmbH, Germany). RT-PCR assays were performed in duplicates using Applied Biosystems 7500 FAST 96-well PCR machine (USA). In order to evaluate the effects of telmisartan on visceral adipose tissue in diet induced obesity hypertension, the mRNA expression levels of the aforementioned genes were measured as described previously [35–37]. Briefly, following homogenization of either fresh or frozen rat visceral adipose tissue samples by TRI reagent (Sigma-Aldrich, UK), total RNA was extracted. Reverse transcription of the rat visceral adipose tissue RNA was done via high-capacity RNA-to-cDNA kit (Applied Biosystems, CA, USA). After That, mRNA expression for the target gene was measured using the generated cDNA. Relative quantity of mRNA expression of the gene of interest was calculated applying the comparative Ct (2−ΔCt) method using β-actin as a control. Data were expressed as the mean ratio (target gene/β-actin%) ± standard error of mean of at least three independent experiments.

### Statistical analysis

Initially, all data were checked of normality using Kolmogorov–Smirnov test. The Analyses of Variances (ANOVA), with Tukey’s post hoc tests were applied. Results were expressed as mean ± standard error (SE), and p values < 0.05 were considered significant. The Origin^®^ software was used for the statistical analysis of data.

## Results

**Fig. 1:**
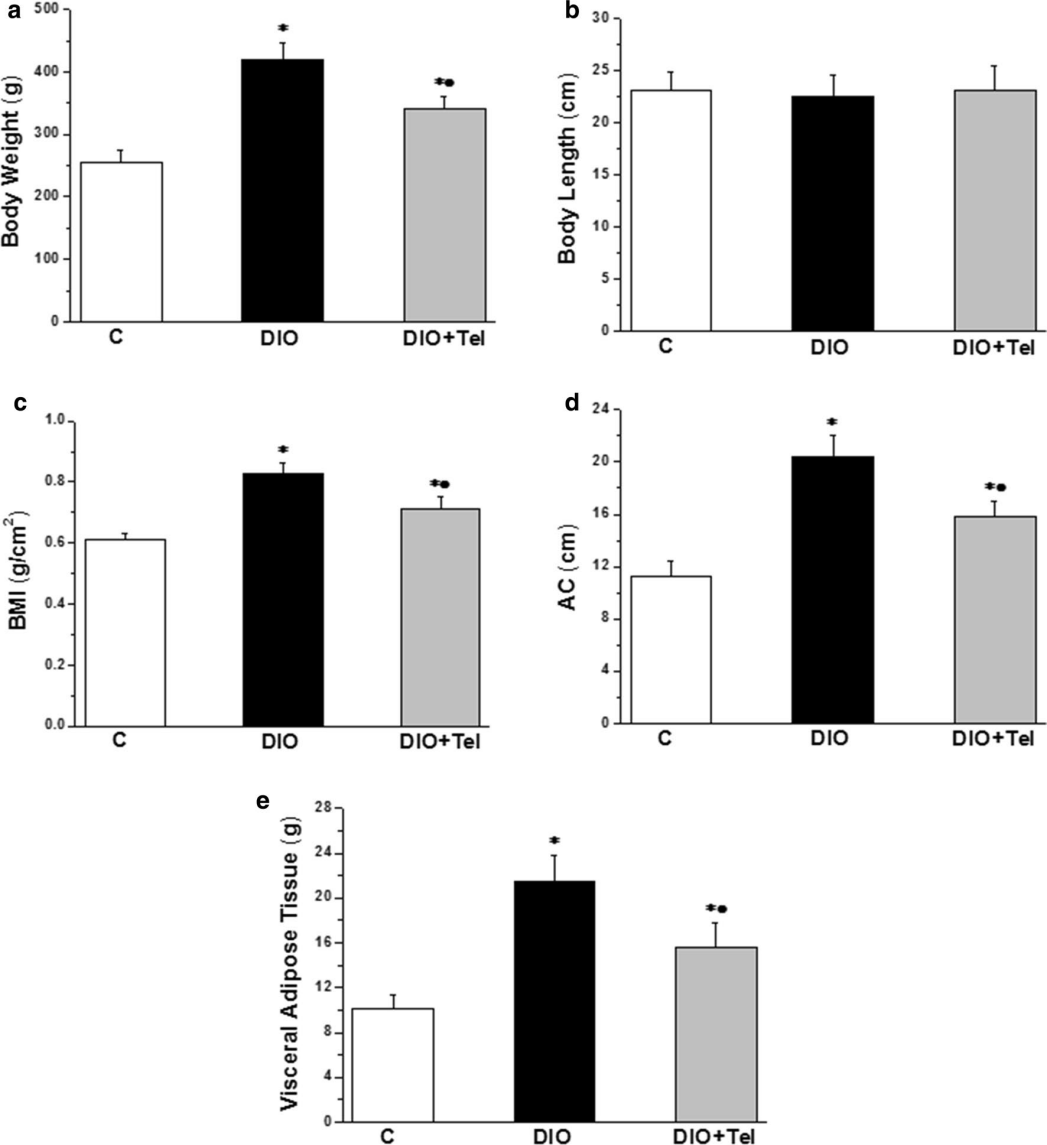
Telmisartan improves altered anthropometric parameters and visceral adipose tissue content in diet-induced obesity hypertension. **a** Body weight in control, DIO and DIO + Tel groups. **b** Body length in control, DIO and DIO + Tel groups. **c** Body mass index in control, DIO and DIO + Tel groups. **d** Abdominal circumference in control, DIO and DIO + Tel groups. **e** Visceral adipose tissue in control, DIO and DIO + Tel groups (number of rats = 10/group. P < 0.05 considered significant, * significant when compared to the control group, • significant when compared to the DIO group)

Body weight increased significantly in the DIO group when compared to the control group (419.22 ± 26.32 vs 255.62 ± 18.24 g, P < 0.05). The body weight decreased significantly in the DIO + Tel group when compared to the DIO group (341.21 ± 19.34 vs 419.22 ± 26.32 g, P < 0.05), while remained significantly higher than the corresponding value in the control group (Fig. 1a). There was no significant difference in the body length among the studied groups (P > 0.05, Fig. 1b). Body mass index (BMI) increased significantly in the DIO group when compared to the control group (0.83 ± 0.03 vs 0.61 ± 0.02 g/cm^2^, P < 0.05). The BMI decreased significantly in the DIO + Tel rats when compared to the DIO rats (0.71 ± 0.04 vs 0.83 ± 0.03 g/cm^2^, P < 0.05). However, it was significantly higher when compared to the control rats (Fig. 1c). Abdominal circumference (AC) increased significantly in the DIO group when compared to the control group (20.42 ± 1.63 vs 11.31 ± 1.08 cm, P < 0.05). AC decreased significantly in the DIO + Tel group when compared to the DIO group (15.78 ± 1.19 vs 20.42 ± 1.63 cm, P < 0.05), but remained significantly increased if compared to the control group (Fig. 1d). Visceral fat mass increased significantly in the DIO group when compared to the control group (21.47 ± 2.37 vs 10.14 ± 1.22 g, P < 0.05). Visceral fat mass decreased significantly in the DIO + Tel group when compared to the DIO group (15.62 ± 2.19 vs 21.47 ± 2.37 g, P < 0.05), but it was still significantly higher than the corresponding values in the control (Fig. 1e).

**Fig. 2:**
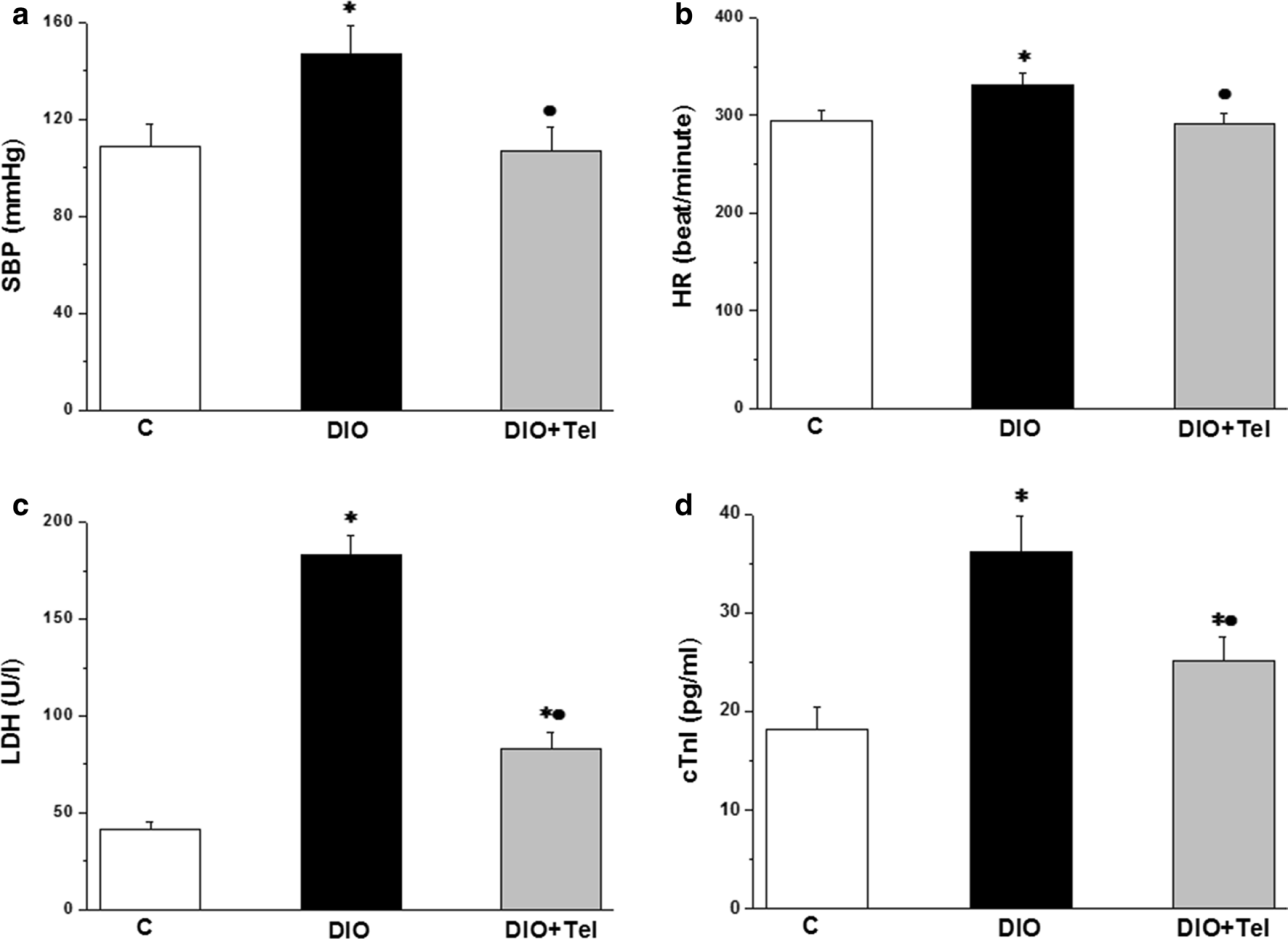
Effect of telmisartan on systolic blood pressure, heart rate and serum levels of cardiac enzymes in diet-induced obesity hypertension. **a** Systolic blood pressure in control, DIO and DIO + Tel groups. **b** Heart rate in control, DIO and DIO + Tel groups. **c** Serum lactate dehydrogenase level in control, DIO and DIO + Tel groups. **d** Serum cardiac troponin I level in control, DIO and DIO + Tel groups (number of rats = 10/group. P < 0.05 considered significant, * significant when compared to the control group, • significant when compared to the DIO group)

Systolic blood pressure (SBP) was significantly higher in the DIO rats when compared to the control rats (147.19 ± 11.35 vs 108.48 ± 9.27 mmHg, P < 0.05). SBP was significantly lower in the DIO + Tel rats when compared to the DIO rats (106.81 ± 10.11 VS 147.19 ± 11.35 mmHg, P < 0.05). There was no statistically significant difference in SBP between the DIO + Tel and the control groups (P > 0.05, Fig. 2a). The heart rate (HR) was significantly higher in the DIO rats when compared to the control rats (331.45 ± 11.19 vs 294.62 ± 10.33 beat/min, P < 0.05). HR was significantly lower in the DIO + Tel rats when compared to the DIO rats (290.87 ± 11.82 vs 331.45 ± 11.19 beat/min, P  < 0.05), while there was no significant difference in the HR between the DIO + Tel and the control groups (P > 0.05, Fig. 2b). Serum LDH and cardiac troponin I (cTnI) levels were significantly higher in the DIO group when compared to the control group (183.24 ± 10.15 U/l and 36.19 ± 3.7 pg/ml vs 41.62 ± 4.13 U/l, and 18.23 ± 2.32 pg/ml respectively, P < 0.05). The serum levels of LDH and cTnI decreased significantly in the DIO + Tel rats (82.79 ± 9.11 U/l and 25.11 ± 2.41 pg/ml respectively, P < 0.05) when compared to the DIO rats, while they were significantly higher that the corresponding values in the control rats (Fig. 2c, d).

**Fig. 3:**
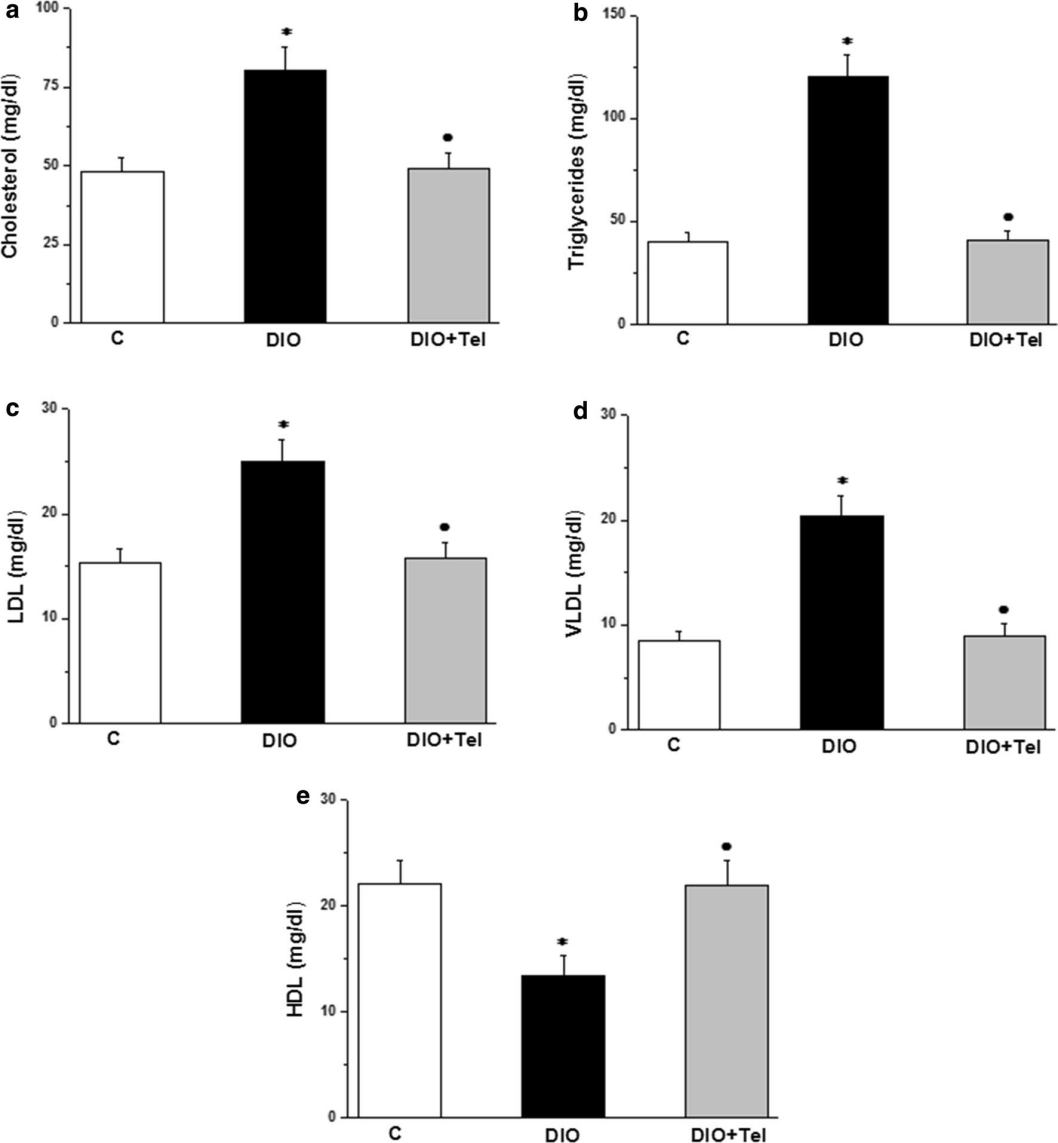
Effect of telmisartan on lipid profile in diet-induced obesity hypertension. **a** Serum total cholesterol level in control, DIO and DIO + Tel groups. **b** Serum triglycerides level in control, DIO and DIO + Tel groups. **c** Serum low density lipoproteins level in control, DIO and DIO + Tel groups. **d** Serum very low density lipoproteins level in control, DIO and DIO + Tel groups. **e** Serum high density lipoproteins level in control, DIO and DIO + Tel groups (number of rats = 10/group. P < 0.05 considered significant, * significant when compared to the control group, • significant when compared to the DIO group)

Serum total cholesterol, triglycerides, low density lipoproteins (LDL), very low-density lipoproteins (VLDL) were significantly higher, while serum high density lipoproteins (HDL) level was significantly lower in the DIO group when compared to the control group (80.11 ± 7.63, 120.19 ± 10.44, 24.96 ± 2.11, 20.38 ± 1.98 and 13.42 ± 1.91 vs 48.12 ± 4.57, 40.31 ± 4.37, 15.33 ± 1.37, 8.44 ± 0.9 and 22.11 ± 2.21 mg/dl respectively, P < 0.05). Serum total cholesterol, triglycerides, LDL, VLDL were significantly lower, while serum high density lipoproteins (HDL) level was significantly higher in the DIO + Tel group (49.33 ± 4.92, 41.28 ± 4.49, 15.79 ± 1.47, 9.03 ± 1.09 and 21.88 ± 2.39 mg/dl respectively, P < 0.05) when compared to the corresponding values in the DIO group. There was no significant difference in serum total cholesterol, triglycerides, LDL, VLDL or HDL when comparing the DIO + Tel and the control groups (P > 0.05, Fig. 3).

**Fig. 4:**
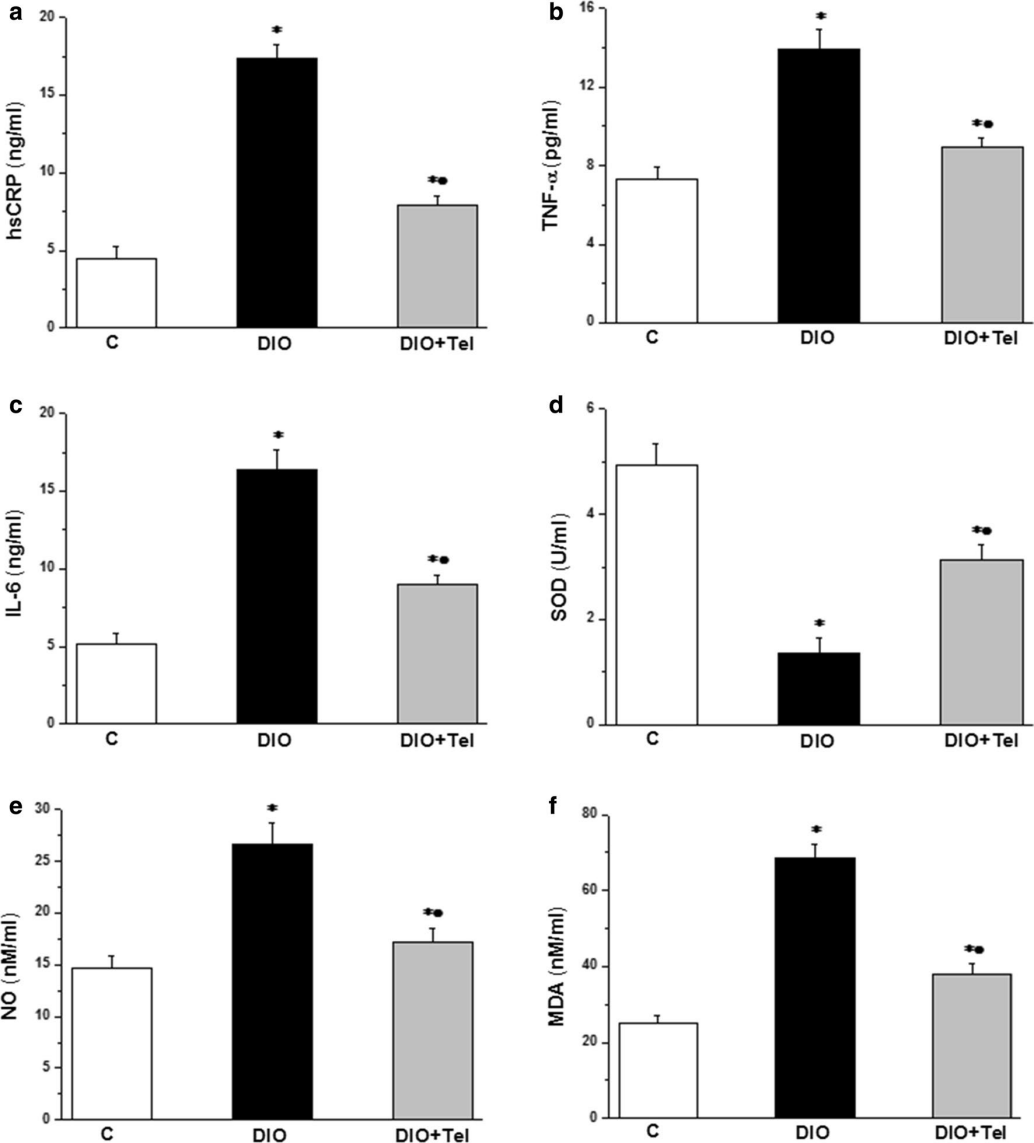
Anti-inflammatory and antioxidative stress effects of telmisartan in diet-induced obesity hypertension. **a** Serum high sensitivity C reactive protein level in control, DIO and DIO + Tel groups. **b** Serum tumour necrosis factor alpha level in control, DIO and DIO + Tel groups. **c** Serum interleukin 6 level in control, DIO and DIO + Tel groups. **d** Serum superoxide dismutase level in control, DIO and DIO + Tel groups. **e** Serum nitric oxide level in control, DIO and DIO + Tel groups. **f** Serum malondialdehyde level in control, DIO and DIO + Tel groups (number of rats = 10/group. P < 0.05 considered significant, * significant when compared to the control group, • significant when compared to the DIO group)

Serum levels of hsCRP, TNF-α and IL-6 were significantly increased in the DIO rats when compared to the control rats (17.37 ± 0.83 ng/ml, 13.89 ± 1.03 pg/ml and 16.38 ± 1.29 ng/ml vs 4.46 ± 0.79 ng/ml, 7.32 ± 0.61 pg/ml and 5.12 ± 0.74 ng/ml respectively, P < 0.05). Serum hsCRP, TNF-α and IL-6 levels decreased significantly in the DIO + Tel rats (7.92 ± 0.53 ng/ml, 8.93 ± 0.52 pg/ml and 8.94 ± 0.63 ng/ml respectively, P < 0.05), when compared to the DIO rats. Serum hsCRP, TNF-α and IL-6 levels remained significantly higher in the DIO + Tel group when compared to the control group (P < 0.05, Fig. 4a–c). Serum SOD decreased significantly in the DIO group when compared to the control group (1.37 ± 0.28 vs 4.95 ± 0.39 U/ml, P < 0.05). Serum SOD increased significantly in the DIO + Tel group (3.14 ± 0.29 U/ml, P < 0.05) when compared to the DIO group, while it was still significantly lower when compared to the corresponding values in the control group (Fig. 4d). Serum levels of NO and MDA increased significantly in the DIO group when compared to the control group (26.59 ± 2.11 and 68.72 ± 3.42 vs 14.63 ± 1.29 and 24.85 ± 2.39 nM/ml respectively, P < 0.05). NO and MDA levels were significantly lower in the DIO + Tel rats (17.13 ± 1.35 and 37.94 ± 2.69 nM/ml respectively, P < 0.05) when compared to the DIO rats, while they were still significantly higher when compared to the corresponding values in the control group (Fig. 4e, f).

**Fig. 5:**
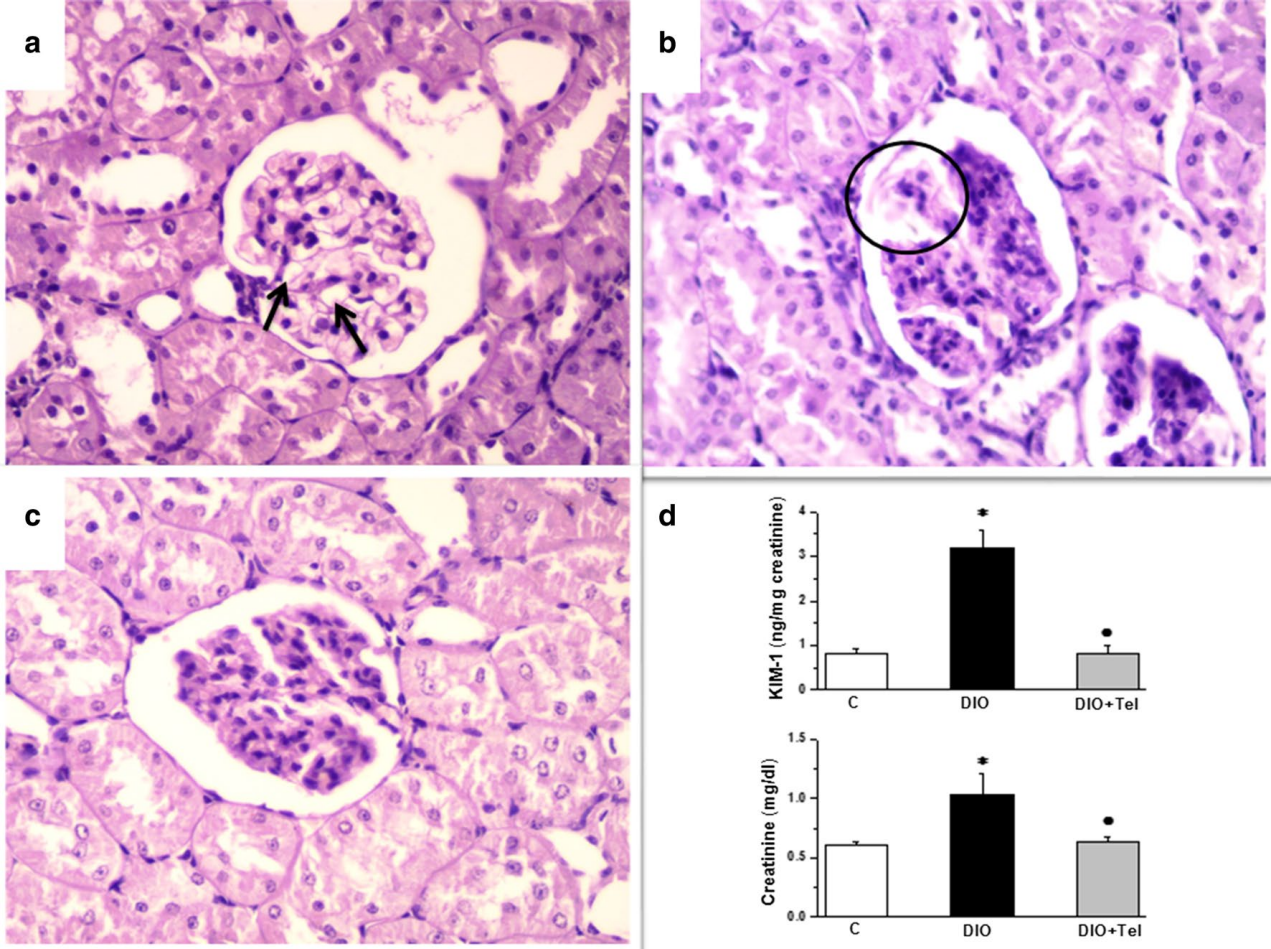
Telmisartan improves diet-induced renal structural and functional damage. **a** Representative photomicrograph of PAS-stained kidney section from the control group showed unremarkable pathological changes with patent glomerular capillary lumina (arrows). **b** Representative photomicrograph of PAS-stained kidney sections from the DIO group showed enlarged glomeruli with preserved Bowman’s space. Accentuation of lobular architecture, hypercellularity, compressed glomerular capillary lumen (circle), mesangial proliferation, and focal mesangial hyalinization were also noticed. **c** Representative photomicrograph of PAS-stained kidney sections from the DIO + Tel group showed mildly enlarged glomerulus with patent Bowman’s space. Subtotal collapse of glomerular capillary lumen and mild mesangial proliferation (PAS  ×400 for **a**, **b** and **c**). **d** Serum kidney injury molecule 1 (upper panel) and creatinine (lower panel) levels in control, DIO and DIO + Tel groups (number of rats = 10/group. P < 0.05 considered significant, * significant when compared to the control group, • significant when compared to the DIO group)

Histopathological evaluation of PAS-stained sections from the kidneys of control group demonstrated normal glomerulus with patent Bowman’s space, normal cellularity, and patent glomerular capillary lumen. There was no mesangial proliferation or mesangial matrix expansion (Fig. 5a). PAS-stained sections from the kidneys of the DIO group showed enlarged glomeruli with preserved Bowman’s space. Accentuation of lobular architecture, hypercellularity, compressed glomerular capillary lumen, mesangial proliferation, and focal mesangial hyalinization were also noticed (Fig. 5b). PAS-stained sections from the kidneys of the DIO + Tel group showed mildly enlarged glomerulus with patent Bowman’s space. Subtotal collapse of glomerular capillary lumen and mild mesangial proliferation could be seen (Fig. 5c). Renal function biomarkers were assessed. Serum KIM-1 and creatinine levels were significantly higher in the DIO group when compared to the control group (3.19 ± 0.39 ng/mg creatinine and 1.03 ± 0.18 mg/dl vs 0.81 ± 0.12 ng/mg creatinine and 0.61 ± 0.03 mg/dl respectively, P < 0.05). Serum levels of both KIM-1 and creatinine were significantly lower in the DIO + Tel rats (0.83 ± 0.15 ng/mg creatinine and 0.64 ± 0.04 mg/dl respectively, P < 0.05), when compared to the DIO rats. There was no significant difference in serum creatinine or KIM-1 when comparing the DIO + Tel and the control groups (P > 0.05, Fig. 5d).

**Fig. 6:**
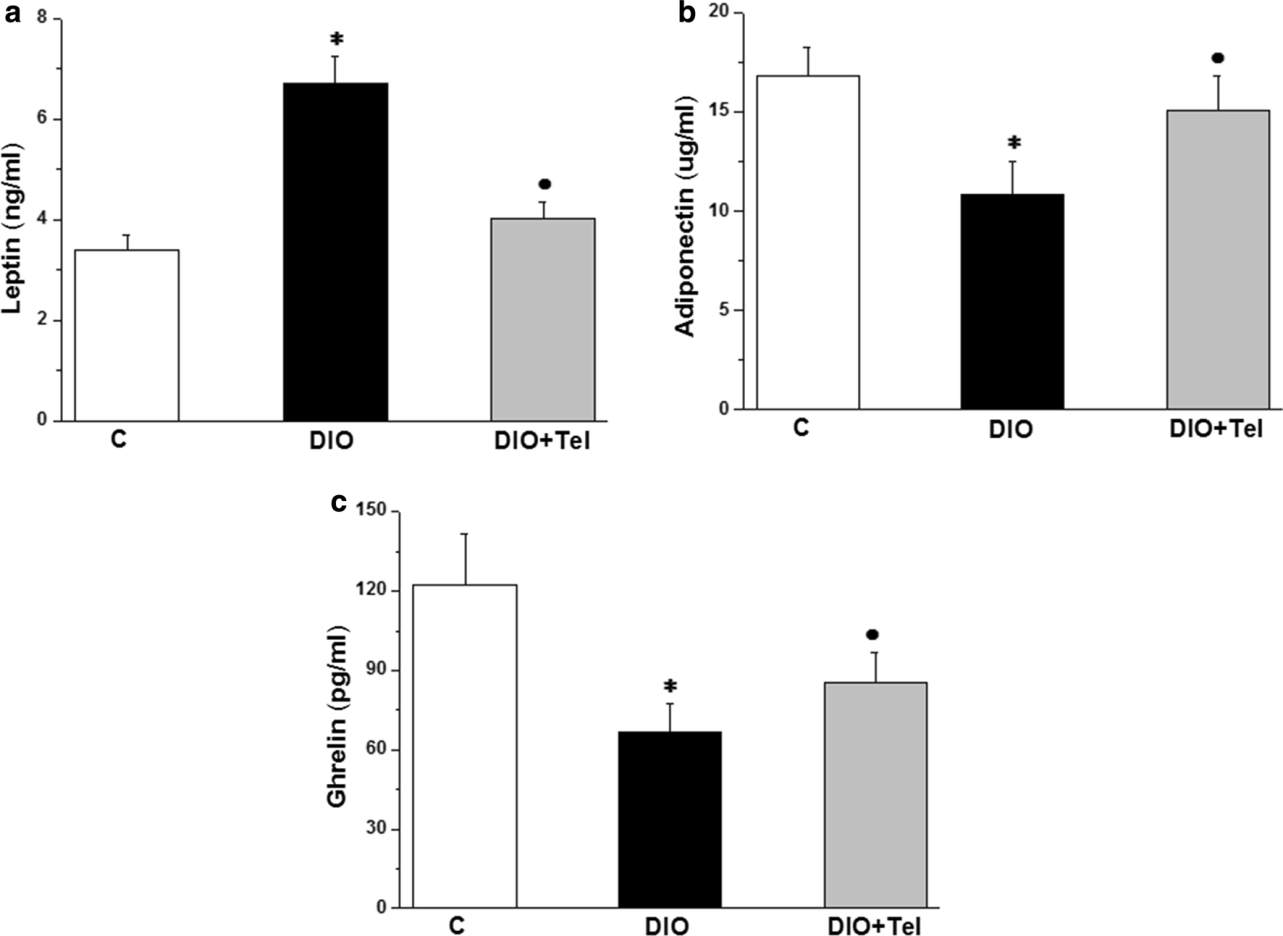
Effect of telmisartan on adipogenicity parameters in diet-induced obesity hypertension. **a** Serum leptin level in control, DIO and DIO + Tel groups. **b** Serum adiponectin level in control, DIO and DIO + Tel groups. **c** Serum ghrelin level in control, DIO and DIO + Tel groups (number of rats = 10/group. P < 0.05 considered significant, * significant when compared to the control group, • significant when compared to the DIO group)

Serum leptin increased significantly in the DIO group when compared to the control group (6.71 ± 0.52 vs 3.41 ± 0.29 ng/ml, P < 0.05). Serum leptin decreased significantly in the DIO + Tel group (4.01 ± 0.34 ng/ml, P < 0.05), when compared to the DIO group. There was no significant difference in serum leptin when comparing the DIO + Tel and the control groups (P > 0.05, Fig. 6a). Serum adiponectin and ghrelin levels were significantly lower in the DIO group when compared to the control group (10.82 ± 1.74 μg/ml and 66.91 ± 10.32 pg/ml vs 16.82 ± 1.44 μg/ml and 122.23 ± 19.37 pg/ml respectively, P < 0.05). Serum adiponectin and ghrelin increased significantly in the DIO + Tel rats when compared to the DIO rats (15.12 ± 1.74 μg/ml and 85.41 ± 11.12 pg/ml respectively, P < 0.05). There was no significant difference in serum levels of adiponectin and ghrelin between the DIO + Tel and the control groups (P > 0.05, Fig. 6b, c).

**Fig. 7:**
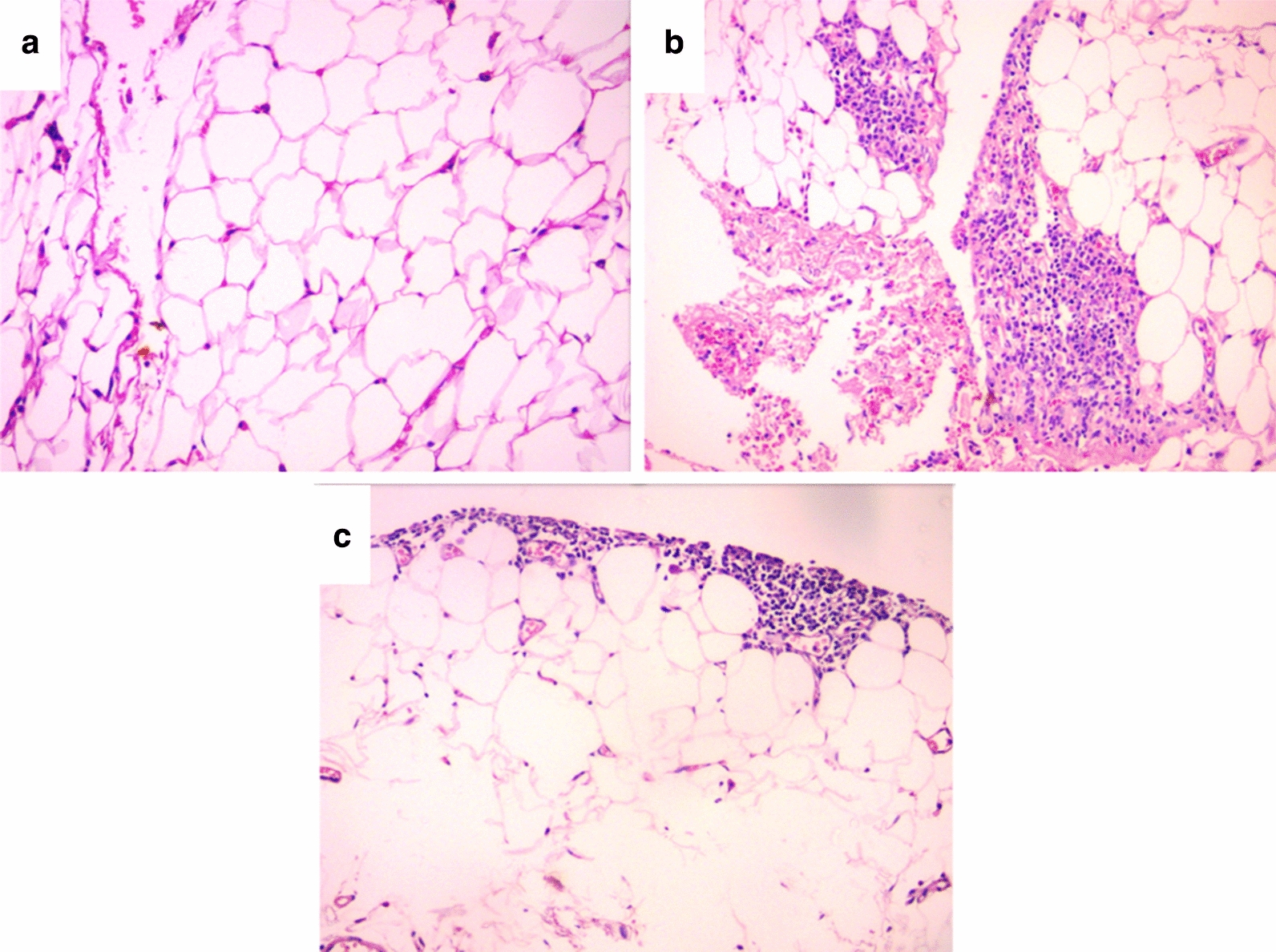
Effect of telmisartan on visceral adipose tissue in diet-induced obesity hypertension. **a** Section of the visceral adipose tissue in the control group showed uniform adipocytes with unremarkable pathological changes. **b** Section of the visceral adipose tissue in the DIO group showed dense mononuclear inflammatory cellular infiltration. **c** Section of the visceral adipose tissue in the DIO + Tel group showed reduction of density of inflammatory cellular infiltrates (H&E ×200 for **a**, **b** and **c**) (number of rats = 10/group. P < 0.05 considered significant, * significant when compared to the control group, • significant when compared to the DIO group)

H&E-stained sections from visceral adipose tissue of the control group demonstrated regular and even white adipocytes. There were no brown adipocytes, lipoblasts, inflammation, congestion, or granuloma (Fig. 7a). H&E-stained sections from visceral adipose tissue of the DIO group showed moderate variability in the size of the white adipocytes (small sized adipocytes and large distended hypertrophied one), congested blood vessels, and intense diffuse chronic inflammatory cellular infiltrates including lymphocytes, mast cells and eosinophils (Fig. 7b). H&E-stained sections from visceral adipose tissue of the DIO + Tel group showed mild variability in adipocytes size (small sized adipocytes and slightly larger and less distended larger one), and mild scattered chronic inflammatory cellular infiltrates (Fig. 7c).

**Fig. 8:**
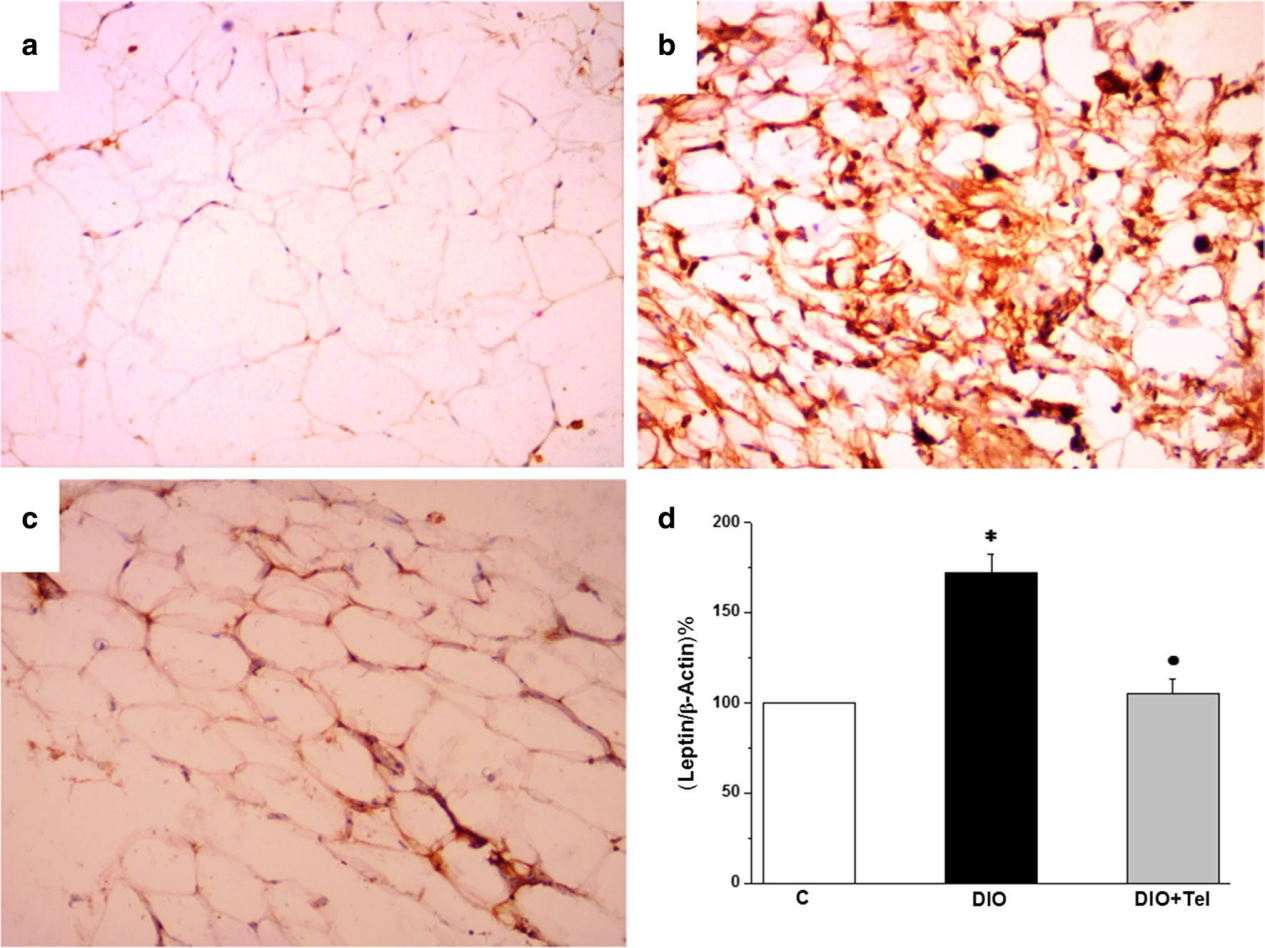
Effect of telmisartan on visceral adipose tissue leptin content in diet-induced obesity hypertension. **a** Section of the visceral adipose tissue in the control group showed faint diffuse cytoplasmic leptin expression. **b** Section of the visceral adipose tissue in the DIO group showed strong diffuse cytoplasmic leptin expression. **c** Section of the visceral adipose tissue in the DIO + Tel group showed mild diffuse cytoplasmic leptin expression (Leptin IHC  ×200 for **a**, **b** and **c**). **d** Leptin mRNA gene expression level in control, DIO and DIO + Tel groups (number of rats = 10/group. P < 0.05 considered significant, * significant when compared to the control group, • significant when compared to the DIO group)

Immunostaining evaluation of sections from visceral adipose tissue of the control group stained with leptin antibody demonstrated faint cytoplasmic leptin immunostaining (Fig. 8a). Sections from the DIO group showed strong diffuse leptin immunostaining and inflammatory cell infiltration (Fig. 8b). Interestingly, sections from the DIO + Tel group showed faint cytoplasmic leptin immunostaining (Fig. 8c). Leptin mRNA gene expression was significantly higher in visceral adipose tissue of the DIO rats when compared to the control rats (172.33 ± 10.21 vs 100 leptin/β-actin%, P < 0.05). Leptin mRNA gene expression in visceral adipose tissue of the DIO + Tel rats was significantly lower (105.16 ± 8.47 leptin/β-actin%, P < 0.05), when compared to the DIO rats. There was no significant difference (P > 0.05) in leptin mRNA gene expression in the visceral adipose tissue between the DIO + Tel and the control groups (Fig. 8d).

**Fig. 9:**
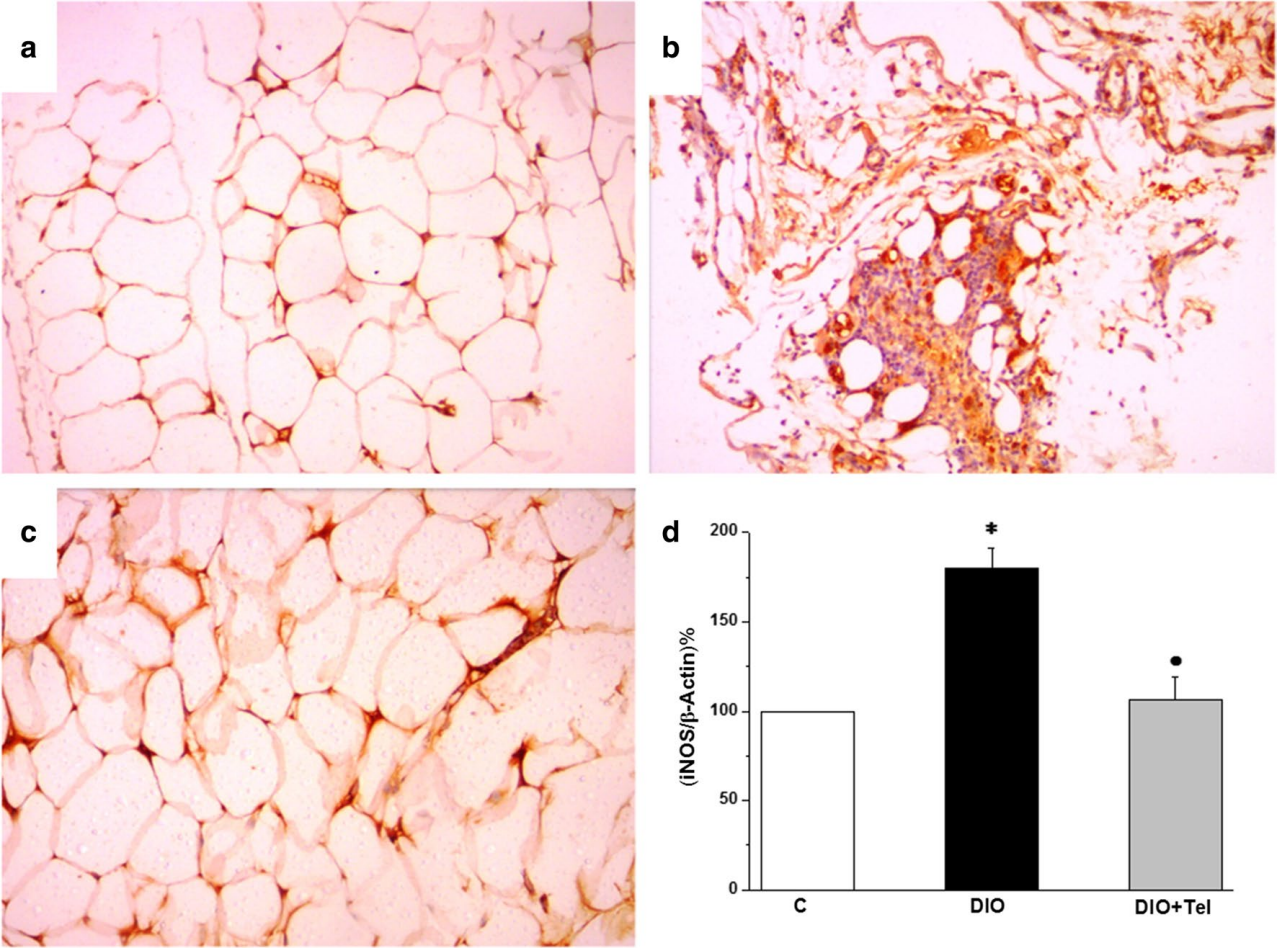
Effect of telmisartan on visceral adipose tissue inducible nitric oxide synthase content in diet-induced obesity hypertension. **a** Section of the visceral adipose tissue in the control group showed mild diffuse cytoplasmic iNOS immunoreactivity. **b** Section of the visceral adipose tissue in the DIO group showed intense diffuse cytoplasmic iNOS immunoreactivity. **c** Section of the visceral adipose tissue in the DIO + Tel group showed moderate diffuse cytoplasmic iNOS immunoreactivity (iNOS IHC ×200 for **a**, **b** and **c**). **d** iNOS mRNA gene expression level in control, DIO and DIO + Tel groups (number of rats = 10/group. P < 0.05 considered significant, * significant when compared to the control group, • significant when compared to the DIO group)

Sections from visceral adipose tissue of the control group stained with iNOS antibody demonstrated mild cytoplasmic diffuse iNOS immunostaining (Fig. 9a). Comparable sections from the DIO group showed strong diffuse iNOS immunostaining and inflammatory cell infiltration (Fig. 9b). Sections from the DIO + Tel group showed moderate cytoplasmic diffuse iNOS immunostaining (Fig. 9c). iNOS mRNA gene expression was significantly higher in visceral adipose tissue of the DIO rats when compared to the control rats (179.91 ± 11.32 vs 100 iNOS/β-actin%, P < 0.05). iNOS mRNA gene expression in visceral adipose tissue of the DIO + Tel rats was significantly lower (106.154 ± 12.83 iNOS/β-actin%, P < 0.05), when compared to the DIO rats. There was no significant difference (P > 0.05) in iNOS mRNA gene expression in the visceral adipose tissue between the DIO + Tel and the control groups (Fig. 9d).

**Fig. 10:**
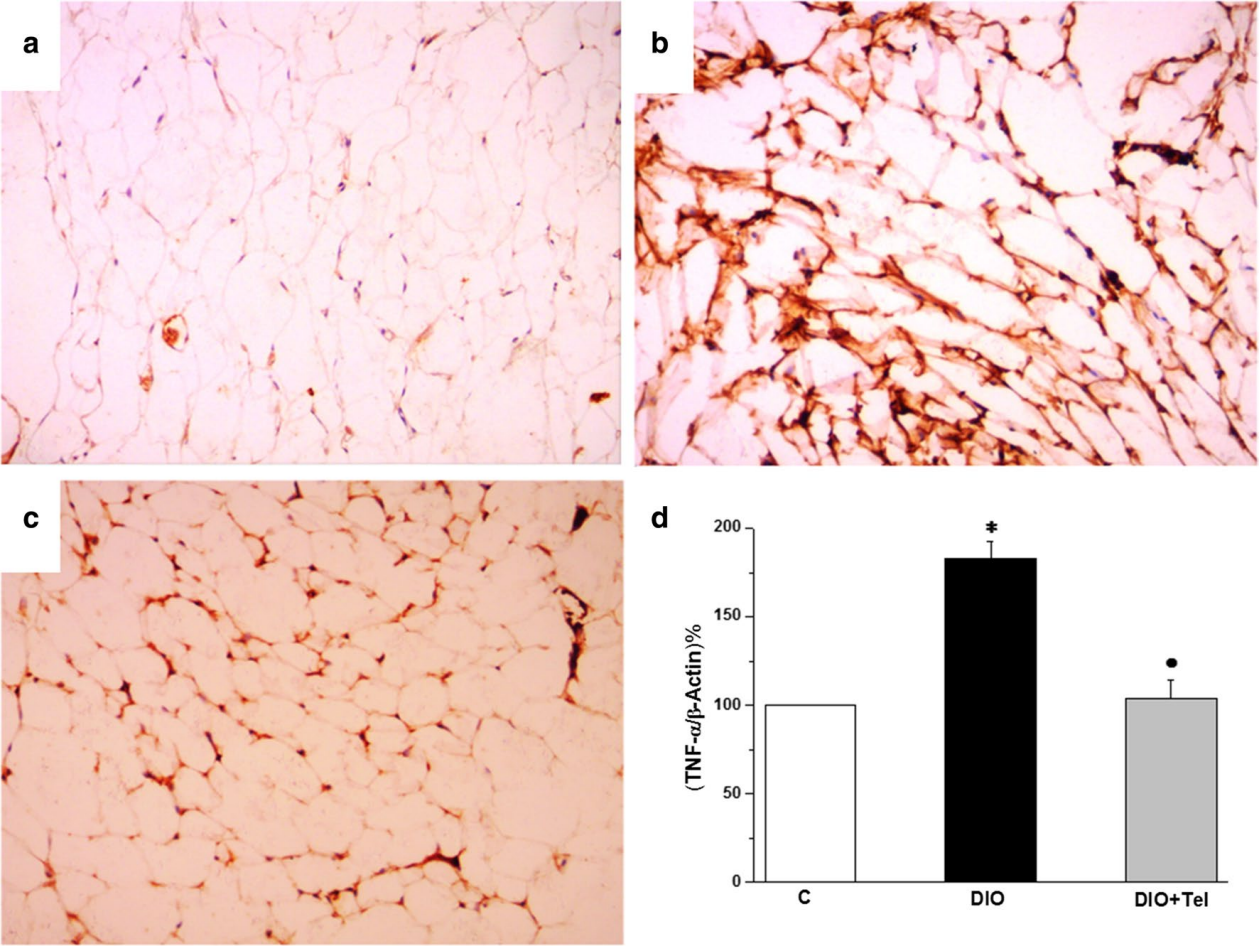
Effect of telmisartan on visceral adipose tissue TNFα content in diet-induced obesity hypertension. **a** Section of the visceral adipose tissue in the control group showed mild diffuse cytoplasmic TNFα immunoreactivity. **b** Section of the visceral adipose tissue in the DIO group showed strong diffuse cytoplasmic TNFα immunoreactivity. **c** Section of the visceral adipose tissue in the DIO + Tel group showed mild to moderate diffuse cytoplasmic TNFα immunoreactivity (TNFα IHC ×200 for **a**, **b** and **c**). **d** TNFα mRNA gene expression level in control, DIO and DIO + Tel groups (number of rats = 10/group. P < 0.05 considered significant, * significant when compared to the control group, • significant when compared to the DIO group)

TNF-α immunostaining in visceral adipose tissue of the control group revealed mild diffuse cytoplasmic staining (Fig. 10a). Comparable sections from the DIO group showed strong diffuse TNF-α cytoplasmic immunostaining (Fig. 10b). Sections from the DIO + Tel group showed mild cytoplasmic diffuse TNFα immunostaining (Fig. 10c). TNF-α gene expression was significantly higher in visceral adipose tissue of the DIO rats when compared to the control rats (183.07 ± 9.15 vs 100 TNFα/β-actin%, P < 0.05). TNFα mRNA level was significantly lower in visceral adipose tissue of the DIO + Tel rats (103.74 ± 10.19 TNF-α/β-actin%, P < 0.05), when compared to the DIO rats. There was no significant difference (P > 0.05) in TNF-α mRNA gene expression in the visceral adipose tissue between the DIO + Tel and the control groups (Fig. 10d).

## Discussion

Obesity has been considered as a major health problem affecting incrementally the global population. The world health organization (WHO) estimates that almost 2 billion adults aged 18 years and older are overweight, with nearly one third of them are obese. The dramatic increase in the prevalence of obesity carries the risk that the majority of adults by 2030 will be either overweight or obese [38]. Undoubtingly, obesity is a major risk factor for essential hypertension. The Results of the Framingham Heart Study suggested that approximately 65% to 75% of the risk for hypertension can be directly linked to excess obesity [39]. Although the association between obesity and hypertension is well established, the exact mechanism, or mechanisms, by which obesity influences hypertension has/have not been clearly established [40]. Obesity-induced hypertension develops via multiple, and may be complex, mechanisms including adipokine alterations, and structural and functional abnormalities in the kidneys [13]. In the present study, we investigated the effect of an angiotensin receptor blocker (ARB) and a peroxisome proliferator activated receptor gamma (PPAR-γ) partial agonist; telmisartan, on diet induced obesity hypertension. We also explored in depth to reveal the possible underlying adipose tissue-related metabolic and endocrinal mechanisms.

### Telmisartan improved altered anthropometric measures and central fat deposition

Studying obesity-related hypertension, or any other comorbidities, require precise definition of obesity in the laboratory animal based on accurate parameters [30]. In this study, high-fat diet induced obesity with the classical alterations in anthropometrical parameters. Total body weight, BMI and abdominal circumference were significantly increased in the diet-induced obesity group, while significantly decreased in the diet-induced obese rats treated with telmisartan. There was no significant difference in the body length between the studied groups, denoting that the changes in body weight, BMI and AC were due to accumulation of fat. We were interested in studying the visceral adipose tissue, since central obesity carries the highest cardiovascular risk [41]. Visceral adipose tissue was significantly increased in diet-induced obesity group. Telmisartan decreased significantly central fat deposition.

### Effect of telmisartan on altered cardiac function and dyslipidaemia

In the present study, high fat diet induced increase in the SBP and HR. The cardiac enzymes, LDH and cTnI, were also elevated in the diet-induced obesity group. Telmisartan showed a cardioprotective effects in terms of decreasing the SBP, normalizing the HR, and reducing the levels of the cardiac enzymes. Obesity is an independent risk factor for cardiovascular disease (CVD). Obesity is also considered as one of the chief risk factors of cardiovascular related disorders such as hypertension, dyslipidemia, atherosclerosis and insulin resistance [42]. Obesity-related hypertension could be due to multiple mechanisms including, endothelial dysfunction, renal affection, stimulation of the renin–angiotensin–aldosterone system (RAAS), and insulin resistance [40]. Increased HR and its variability are common in obesity, while it decreases in obese-prediabetic and obese-diabetic individuals [9, 40, 43]. One of the grave outcomes of obesity, is the subclinical elevation of cardiac enzymes including troponin and lactate dehydrogenase [44]. Obesity is then rendering the heart prone to failure, and therefore, increasing cardiovascular mortality. Telmisartan has been reported to counter alteration in BP and HR in different experimental and clinical settings [20, 45, 46]. In a recent study, telmisartan decreased cTnI in dogs subjected to experimental supraventricular tachyarrhythmia [47]. The complexity of obesity-dependent cardiovascular changes raises the possibility that telmisartan could be acting via multiple mechanisms and not only via blocking angiotensin II receptors.

In our study, telmisartan was able to prevent obesity-induced dyslipidemia. The link between dyslipidemia and obesity is extensively studied. The most common obesity-associated dyslipidemias include elevated levels of triglycerides and LDL, and decreased level of HDL [48]. In obesity, most of the ingested excess energy is stored within adipocytes in the form of triglycerides, which are formed through the binding of glycerol and fatty acids [49]. The term dyslipidemic hypertension (DH) has gained attention in the last 4 decades, owing to the prime role of their comorbidity in cardiovascular pathophysiology [50]. The Saga Telmisartan Aggressive Research (STAR), a single-arm, prospective multi-center trial, evaluated the effectiveness of telmisartan to lower blood cholesterol in dyslipidemic hypertensive patients, and reported the potentiality of telmisartan in the treatment of hypertension-related dyslipidemia [51]. Moreover, telmisartan was thought to be superior to other ARBs in reducing blood triglycerides level [52]. In an experimental study on streptozotocin diabetic rats, telmisartan was capable to increase HDL while it decreased VLDL levels [53]. Our data support the potential superiority of telmisartan in the treatment of obese hypertensive patients.

### Anti-inflammatory and antioxidant effects of telmisartan

In the present study, obesity resulted in systemic inflammation and oxidative stress. Intriguingly, telmisartan had a profound anti-inflammatory and antioxidant effects in obese hypertensive rats. In the broader sense, obesity and hypertension have both the inflammatory and the oxidative stress facets [54, 55]. Serum CRP and IL-6 were significantly higher in hypertensive patients when compared to normotensive counterparts [54]. Telmisartan monotherapy significantly decreased serum levels of CRP and IL-8 in obese hypertensive patients [46]. Interestingly, telmisartan had a potential anti-inflammatory effect in the treatment of anaemia of chronic disease in a rat model of arthritis. In that study, telmisartan significantly reduced serum levels of TNF-α and IL-6 levels [56]. The antioxidant properties of telmisartan have been demonstrated recently in different settings. In a rat skeletal muscle ischemia reperfusion study, telmisartan enhanced the SOD and catalase (CAT) scavenging activities, and in parallel, decreased the oxidative products [57]. The neuroprotective effect of telmisartan in a setting of high glucose (HG)-elicited oxidative damage in PC12 cells was mediated via PPAR-γ activation and included elevation of SOD and CAT levels and decrease in MDA level [58]. Attenuating lipid peroxidation and increasing the endogenous antioxidant protein glutathione were also noticed when treating acute doxorubicin-induced cardiotoxicity with telmisartan [59]. Our study could be one of few demonstrating the potential effects of telmisartan on systemic inflammation and oxidative stress in obesity-related hypertension.

### Preservation of kidney structure and function

This study demonstrated that obesity exerted both structural and functional renal changes, compromising the renal functions in obesity. As expected, administration of an ARB preserved the renal functions and assumably enabled the kidneys to correct the obesity-related elevation in arterial blood pressure. The high fat dietary model of obesity almost mimics the metabolic, neurohumoral, renal, and cardiovascular changes observed in obese humans. These changes include elevation in arterial blood pressure, increased heart rate, activation of the renin–angiotensin–aldosterone system (RAAS), stimulation of the sympathetic nervous systems, sodium and water retention, and expansion of the extracellular fluid volume [60, 61]. Our results were in agreement with previously published reports which demonstrated that telmisartan could ameliorate metabolic syndrome associated nephropathy by reducing leptin release from the perirenal adipose tissue [62].

### Telmisartan and adipogenicity markers

In the present study, telmisartan opposed obesity-induced alterations in serum adipokines and ghrelin serum levels. This could imply that telmisartan preserves much more of the adipose tissue functions beyond the PPAR-γ dependent lipid metabolic effects. Although leptin was thought to be an anti-obesity hormone owing to its metabolic effects, obese individuals develop leptin resistance disrupting its satiety and weight-reducing effects [63]. Interestingly, leptin resistance spares the leptin-induced activation of the sympathetic nervous system to the kidney, heart, and adrenal glands [64]. Moreover, leptin influences the production of NO, and combined with its stimulatory effect on the renal sympathetic supply, it can result in sodium retention and elevation of the blood pressure [65]. Therefore, leptin should be considered a pivotal player in the development of obesity-related hypertension. In fact, approaches to decrease leptin levels have been shown to be effective in countering obesity and obesity-related comorbidities. For example, spontaneous running decreased the gene expression of leptin mRNA in the visceral and subcutaneous white adipose tissue (WAT) of obese rats [49]. The plasma concentration adiponectin, a plasma protein entirely formed and secreted by adipose tissue, decreased in patients with obesity, coronary heart disease (CHD), and hypertension. Moreover, adiponectin gene mutation has been shown to affect the prevalence of obesity-unrelated clustering of hypertension, diabetes mellitus, and dyslipidemia [66]. Additionally, several studies reported the decrease in serum adiponectin level in animal models of obesity [67]. Interestingly, replenishing adiponectin proved to be a potential strategy in the treatment of obesity-related comorbidities [66]. In the present study, we measured the serum high-molecular-weight adiponectin, which is a more valid marker of cardiovascular risk progression on arterial stiffness [68]. Therefore, the decreased serum level of adiponectin in the diet induced obesity group could refer to an increased arterial stiffness and subsequently explain the rise in systolic blood pressure in that group. Telmisartan was reported to improve adiponectin production in hypertensive patients with type II diabetes mellitus [69]. The association between obesity and hypertension is thought to be deciphered by ghrelin signalling pathway. Consequently, ghrelin could serve as a potential therapeutic target for the management of obesity and hypertension. Ghrelin is known to antagonize the effects of leptin on food intake, weight gain and blood pressure. While the blood level of leptin increases in obesity-related hypertension, the blood level of ghrelin decreases [70]. In a recent study, it was evident that the weight loss-related reduction in BP was mediated via a ghrelin-dependent mechanism [71].

In the present study, diet-induced obesity resulted in alteration in adipocytes morphology representing WAT remodelling. It has been shown before that during positive energy balance adipocyte hypertrophy and hyperplasia may occur because of an increase in triacylglycerols storage. Distension of adipocytes with lipids induced adipose tissue dysfunction in the form of altered adipokine secretion, and adipose tissue inflammation [72]. In our hands, inflammatory changes were evident in the studied sections from the DIO group. Furthermore, serum biomarkers supported the idea of altered inflammatory status in obese rats. Interestingly, telmisartan ameliorated DIO-dependent adipose tissue remodelling, showing both structural, and subsequently functional preservation of adipose tissue function in obesity-related hypertension.

### Gene modifying effects of telmisartan

In the present study, both immunohistochemistry and RT-PCR experiments demonstrated an increase in visceral adipose tissue content of leptin, iNOS and TNF-α in the diet induced obesity group. This effect was attenuated by telmisartan, indicating essential effects of telmisartan on visceral adipose tissue endocrinal functions, oxidant/antioxidant homeostasis, and inflammatory status. Basically, there are three types of adipocytes constituting two distinctive types of adipose tissue. The white adipocytes constitute the main cells of the white adipose tissue. The brown adipocytes are the principal cells of the brown adipose tissue. Recently, beige adipocytes were discovered and found embedded within white adipose tissue [73]. The release of leptin is known to inhibit hunger sensation. Therefore, it is considered as the satiety protein. Ghrelin antagonizes leptin actions, and therefore it is thought to be a hunger hormone [74]. Interestingly, elevated leptin levels have been reported in most obese subjects raising the possibility that chronic elevation of leptin levels diminishes the responses to leptin itself [75]. Hence, and despite the overwhelming stored energy, satiety signals are ignored and more energy resulting from increased food consumption is stored. The higher level of leptin in obese subjects is positively correlated to a higher number of white adipocytes which are believed to be the primary source of leptin [74, 76]. Growing evidence supports the notion that obesity promotes a state of chronic, low-grade inflammation. Although some argued that TNF-α is not systemically released by adipose tissue, the diffuse nature of adipose tissue as well as its close association with metabolically relevant tissues suggests that adipose tissue-derived TNF-α over-production could target non-adipose tissues during obesity. Nevertheless, and due to the improved sensitivity and specificity of testing reagents, circulating soluble TNF-α (sTNF-α) seems convincedly correlated to BMI [77, 78]. Although the exact mechanism is not completely understood, TNF-α has been linked to salt-sensitive hypertension (SSH) and associated renal injury [79]. Interestingly, in our hands there was a significant elevation in the levels oxidative stress biomarkers which were reported to promote TNF-α production. Despite the fact that whether inflammation leads to ROS production with subsequent oxidative stress, or oxidative stress leads to inflammation is quite debatable. WAT inflammation is linked to cellular oxidative stress. Oxidative stress-induced enhancement of iNOS activity results in over production of NO [80]. As shown by the increased serum NO in the present study, nitrosative stress was evident in the diet induced obesity group. Beside its regulatory roles, NO it can react with superoxide anion, forming peroxynitrite, a reactive and oxidant compound able to induce nitration and nitrosation of proteins [81]. The reaction between NO and ROS produces nitrosative stress with a resultant adipocyte dysfunction [80].

Taken all together, our data illustrated that telmisartan cardio- and reno-protective mechanisms could be linked, at least in part, to countering adipose tissue dysfunction. A possible anti-inflammatory and antioxidant mechanisms may underlie telmisartan potential effects in the treatment of obesity hypertension, beyond its angiotensin-blockade and PPAR-γ partial agonist facets. Apparently, telmisartan influences the gene expression level of iNOS, TNF-α and leptin, raising the necessity for elucidating the relevant molecular pathways.

### Study limitations

The most significant limitation of the present work is the observational nature of the study design. Although the aim was to shed the light on a possible central role of adipose tissue dysfunction in the obesity-hypertension dilemma, more work is necessary to revel the pathways responsible for the alteration of adipose tissue function. Knocking down PPAR-γ or angiotensin receptors would have proven that telmisartan effects could be also mediated via different mechanisms such as the anti-inflammatory and antioxidant effects which are proposed in this manuscript.

## Conclusion

Finding the central pathology may decipher the complexity of obesity-related hypertension and achieve better therapeutic outcomes. Adipose tissue dysfunction could be the central pathology of, or at least a major contributor to, obesity related hypertension. Polymodal molecules, such as telmisartan, fit better as an ideal therapeutic tool in the treatment of obese hypertensive patients.

## Data Availability

All the data generated or analysed during this study are included in this published manuscript.

## Abbreviations

AC: Abdominal circumference
ARBs: Angiotensin receptors blockers
AT: Adipose tissue
BP: Blood pressure
BMI: Body mass index
BAT: Brown adipose tissue
CVD: Cardiovascular disease
cTnI: Cardiac troponin I
CAT: Catalase
CHD: Coronary heart disease
DBP: Diastolic
DMSO: Dimethyl sulfoxide
DH: Dyslipidemic hypertension
HR: Heart rate
HDL: High density lipoprotein
hsCRP: High sensitivity C reactive protein
H&E: Haematoxylin and eosin
iNOS: Inducible nitric oxide synthase
IL-6: Interleukin 6
KIM-1: Kidney injury molecule 1
LDH: Lactate dehydrogenase
LDL: Low density lipoprotein
RAAS: Renin–angiotensin–aldosterone system
MDA: Malondialdehyde
NO: Nitric oxide
PAS: Periodic Acid Schiff
PPAR-γ: Peroxisome proliferator-activated receptor gamma
SOD: Superoxide dismutase
SNS: Sympathetic nervous system
SBP: Systolic
TNF-α: Tumour necrosis factor alpha
T2DM: Type 2 diabetes mellitus
VLDL: Very low density lipoprotein
WAT: White adipose tissue
